# Identification and Functional Analysis of *ToBPI1/LBP* and *ToBPI2/LBP* in Anti-Bacterial Infection of *Trachinotus ovatus*

**DOI:** 10.3390/genes14040826

**Published:** 2023-03-29

**Authors:** Ze-Chang Bian, Xiao-Hui Cai, Kian Ann Tan, Ya-Dan Wang, Zhuang Huang, Kit Yue Kwan, Peng Xu

**Affiliations:** 1 Guangxi Key Laboratory of Beibu Gulf Marine Biodiversity Conservation, College of Marine Sciences, Beibu Gulf University, Qinzhou 535011, China; 2 State Key Laboratory for Conservation and Utilization of Subtropical Agro-Bioresources, College of Animal Science and Technology, Guangxi University, Nanning 530004, China

**Keywords:** *Trachinotus ovatus*, gene expression, bactericidal/permeability-increasing protein, bacteriostatic ability

## Abstract

Bactericidal/permeability-increasing protein (BPI) and lipopolysaccharide-binding protein (LBP) are a group of antibacterial proteins that play an important role in the host’s innate immune defense against pathogen infection. In this study, two BPI/LBPs, named *ToBPI1/LBP* (1434 bp in length, 478 amino acids) and *ToBPI2/LBP* (1422 bp in length, 474 amino acids), were identified from the golden pompano. *ToBPI1/LBP* and *ToBPI2/LBP* were significantly expressed in immune-related tissues after being challenged with *Streptococcus agalactiae* and *Vibrio alginolyticus*. The two BPI/LBPs showed significant antibacterial activity against Gram-negative *Escherichia coli* and Gram-positive *S. agalactiae* and *Streptococcus iniae*. In contrast, the antibacterial activity against *Staphylococcus aureus*, *Corynebacterium glutamicum*, *Vibrio parahaemolyticus*, *V. alginolyticus* and *Vibrio harveyi* was low and decreased with time. The membrane permeability of bacteria treated with recombinant ToBPI1/LBP and ToBPI2/LBP was significantly enhanced. These results suggest that ToBPI1/LBP and ToBPI2/LBP may play important immunological roles in the immune response of the golden pompano to bacteria. This study will provide basic information and new insights into the immune response mechanism of the golden pompano to bacteria and the function of BPI/LBP.

## 1. Introduction

Innate immunity is an essential component of the biological immune defense system. It is crucial for maintaining host-microbial homeostasis and for the host’s defense system against the invasion of numerous pathogens such as viruses, bacteria and parasites [1,2,3]. Antimicrobial peptides are a kind of polypeptides with antibacterial activity found in many organisms [4]. There are many kinds of antimicrobial peptides, which can be divided into animal-derived antimicrobial peptides, plant-derived antimicrobial peptides and microbial-derived antimicrobial peptides [5]. Bactericidal/permeability enhancing protein (BPI) and lipopolysaccharide binding protein (LBP) are animal-derived antimicrobial peptides, also members of the lipid transfer/endotoxin binding proteins family; they play an important role in host defense against pathogens and can regulate the innate immune response via pathogen-associated molecular patterns. Structurally and functionally, BPI and LBP are very similar; they own one domain, BPI/LBP/CETP, at the N-terminal that can bind to lipopolysaccharides and one domain, BPI/LBP CETP, at the C-terminal that can transport lipopolysaccharides to other host molecules [2]. However, they also differ significantly in function [6,7]. LBP binds to LPS and enhances the cell response to LPS, whereas BPI binds to Gram-negative bacteria and kills the bacteria by neutralizing LPS and inhibiting the cellular response to LPS [7]. In addition to neutralizing lipopolysaccharide, studies have demonstrated that BPI serves physiological functions such as conditioning, anti-inflammatory and reproduction [8,9]. BPI has currently been shown to have numerous biological functions in mammals. Recombinant mouse BPI, for instance, has been demonstrated to inhibit endotoxin-mediated macrophage activation, while human BPI has been shown to have good antibacterial activity [10]. Additionally, several investigations have also discovered that BPI has a role in activating complement [11]. In teleost fish, BPI or LBP homologues are not classified as BPI or LBP, but they are referred to as BPI/LBP [3]. In fish, BPI/LBP exhibited antibacterial, immunomodulatory and endotoxin-binding properties. An example of this would be the recombinant RfBPI/LBP obtained from *Sebastes schlegelii*, which exhibited antibacterial activity against *E. coli* in vitro [12]. The recombinant MaBPI/LBP protein has shown effective bactericidal activity against *E. coli* and *Aeromonas hydrophila* in *Megalobrama amblycephala* [3]. The recombinant CsBPI protein increases body resistance to bacterial and viral infections in *Cynoglossus semilaevis* by increasing the expression of several genes involved in antibacterial and antiviral immunity [13]. SmBPI/LBP1 demonstrated a high affinity for lipopolysaccharide in *Scophthalmus maximus*, and its binding capacity increased significantly with an increase in protein concentration [14].

Because of its short growth cycle, tasty meat, and high economic value, the golden pompano (*Trachinotus ovatus*) has become a popular mariculture fish in China [15]. The *T. ovatus* mariculture sector has recently suffered significant financial losses as a result of disease outbreaks. Studies have shown that after the golden pompano is infected by a bacteria, immune-related pathways and genes will be activated to participate in the body’s immune response, such as immune-related pathways, the JAK-STAT signaling pathway, Th1 and Th2 cell differentiation, the TNF signaling pathway, the NF-κB signaling pathway and immune-related genes *NF-κB*, *TNF-α*, *IL1*, *IL6*, *IL8*, *IFN-γ* [16]. Wu et al. studied the bacteriostatic effect of golden pompano’s BPI/LBP on Gram-negative bacteria, but its effect on bacterial cell membrane permeability was not studied, and its bacteriostatic effect on Gram-positive bacteria was not clear [17]. Therefore, the function of the golden pompano’s BPI/LBP requires further study. In this study, two BPI/LBP genes, designated as *ToBPI1/LBP* and *ToBPI2/LBP*, were isolated from *T. ovatus*. In addition, we also analyzed the expression, antibacterial activity and cell membrane permeability of *ToBPI1/LBP* and *ToBPI2/LBP* in immune-associated tissues to reveal their immunological function. These results should serve as an important reference for the immune defense and function of *ToBPI1/LBP* and *ToBPI2/LBP*.

## 2. Materials and Methods

### 2.1. Fish and Bacterial Strain

Two hundred individuals of *T. ovatus* with an average weight of 50 ± 0.7 g were purchased from local farms in Qinzhou, Guangxi, China. The fish samples were placed in 200 L of cyclically aerated seawater and kept between 28° and 30° for 10 days. *Streptococcus agalacia*, *V. alginolyticus*, *S. iniae*, *S. aureus*, *C. glutamicum*, *E. coli*, *V. harveyi*, *V. parahaemolyticus* were all donated by Dr. Cai from Beibu Gulf University, Qinzhou, Guangxi, China, whereas *E. coli* DH5α and BL21 were purchased from Beijing TransGen Biotech Co., Ltd. Both *S. agalactiae* and *V. alginolyticus* were cultured in BHI medium overnight at 37°, and then inoculated in 10 mL of liquid medium for shock culture for 10 h. The bacteria were collected, cleaned with 1× PBS three times, and diluted to 1 × 10^7^ CFU/mL for later use.

### 2.2. Fish Challenge Experiments and Tissue Sample

Before dissection, fish were euthanized by MS-222, as previously reported in [18]. Six fish were used to determine the expression of *ToBPI1/LBP* and *ToBPI2/LBP* in healthy tissues. The collected tissues include the heart, gill, stomach, intestine, head, kidney, liver, spleen, brain and muscle. The remaining 190 fish were randomly divided into the control group, *V. alginolytica* group and *S. agalactiae* group, and each fish was intraperitoneally injected with 100 μL PBS or 1 × 10^7^ CFU/mL bacterial suspension, respectively. At 0 h, 6 h, 12 h, 24 h, 36 h, 48 h and 72 h after infection, the tissues, including head, kidney, liver and spleen of six fish in each group, were collected and frozen at −80° until further analysis. In this study, all experiments were conducted under a protocol approved by the Institutional Animal Care and Use Committee (IACUC, China). 

### 2.3. Gene Cloning

All PCR primers used in this study are listed in Table 1. Total RNA was extracted from the spleen using a TRIzol reagent (Invitrogen, USA), and RNA quality was detected by 1% agarose gel, stored at −80 °C for backup. The first strand of cDNA was synthesized according to the manufacturer’s instructions using NovoScript Plus one-step 1st Strand cDNA Synthesis SuperMix (Novoprotein, Shanghai, China). Using the cDNA from the spleen tissue of the golden pompano as the template, the CDS sequences of *ToBPI1/LBP* and *ToBPI2/LBP* were amplified by the following primers, ToBPI1/LBP-F and ToBPI1/LBP-R, ToBPI2/LBP-F and ToBPI2/LBP-R with Premix TaqTM Hot Start Version (TaKaRa, Dalian, China). The reaction system consisted of 4 μL of cDNA, 2 μL of each primer, 24 μL of Premix Taq, and 24 μL of H_2_O. The reaction procedure was run at 95 °C for 30 s; 94 °C for 30 s, 53 °C/57 °C for 30 s, 72 °C for 1 min, for 35 cycles; and at 72 °C for 10 min and 4 °C forever. The amplified product was cloned into the *pEASY*-T1 vector and transformed into *E. coli* DH5α cells. The positive clones were screened and sequenced by the Dongxuan gene (Jiangsu, China).

### 2.4. Bioinformatics Analysis

A similarity analysis and an open reading frame determination were performed through the National Center for Biotechnology Information (NCBI), BLAST and ORF Finder programs. The expert Protein Analysis System (EXPASY) server (http://web.expasy.org/protparam/, accessed on 2 March 2023) was used for molecular weight and theoretical isoelectric point analysis. SignalP 5.0 (https://services.healthtech.dtu.dk/service.php?SignalP-5.0, accessed on 2 March 2023) was performed for the prediction of signal peptides. The Interpro program (http://www.ebi.ac.uk/interpro/, accessed on 2 March 2023) was used for protein domain prediction, and the phylogenetic tree was built using MEGA 7.0.

### 2.5. Expression of ToBPI1/LBP and ToBPI2/LBP in Healthy and Challenged T. ovatus

In this study, the expression patterns of *ToBPI1/LBP* and *ToBPI2/LBP* in the control and experimental groups were evaluated by real-time quantitative polymerase chain reaction, and the β-actin gene was used as an internal control in all RT-qPCR experiments. The specific primers for *ToBPI1/LBP*, *ToBPI2/LBP* and β-actin were designed using the Primer 5.0 software (Table 1). The NovoStart^®^ SYBR qPCR SuperMix Plus Kit (Novoprotein, Shanghai, China) was used to perform a real-time quantitative polymerase chain reaction on the QuantStudioFlex 6Flex real-time fluorescent quantitative polymerase chain reaction system; the reaction system required 0.5 μL of cDNA, 0.4 μL of each primer, 5.2 μL of the SYBR qPCR Mix, and 3.5 μL of H_2_O. The reaction conditions were as follows: predenaturation at 95 °C for 30 s once, retention at 95 °C for 5 s, retention at 56 °C for 15 s, and retention at 72 °C for 10 s for 40 cycles. The expression results were normalized by 2^−△△CT^ and each experiment was repeated three times.

### 2.6. Prokaryotic Expression, Purification and Western Blotting of the Recombinant ToBPI1/LBP(rToBPI1/LBP) and ToBPI2/LBP(rToBPI2/LBP)

To understand the specific biological functions of ToBPI1/LBP and ToBPI2/LBP, open reading frames were amplified with gene-specific primers (mToBPI1/LBP-F andmToBPI1/LBP-R, mToBPI2/LBP-F and mToBPI2/LBP-R), and then cloned into the expression vectors pSumo-mut and pET-32a. Then, the recombinant vectors (pSumo-mut -ToBPI1/LBP and pET-32a-ToBPI2/LBP) were transformed into *E. coli* BL21 (DE3) cells for screening and sequencing. Sequence-correct bacteria were cultured to OD_600_ values of 0.6–0.8 in LB medium containing 20 μg/mL kanamycin and ampicillin. Then, the recombinant strains of rToBPI1/LBP and rToBPI2/LBP were induced by 0.2 mM IPTG for 4 h, and SDS-PAGE was performed for expression analysis of the recombinant protein. The rToBPI1/LBP and ToBPI2/LBP proteins were purified by Ni Seflnose resin under denaturing and renaturation conditions according to our previous work. The recombinant proteins were verified by 12% SDS-PAGE, and protein concentrations were measured by the BCA Protein Assay kit (Beyotime). 

During Western blot analysis, rToBPI1/LBP and rToBPI2/LBP were separated by 12% SDS-PAGE and electrophoretically transferred onto a PVDF membrane. After the transfer, the proteins were washed with PBST 4 times every 5 min, and then the membrane was placed in 5% skim milk powder solution and blocked at 37 °C for 1 h. Anti-his labeled mouse monoclonal antibody was incubated at room temperature for 2 h. After the incubation of the primary antibody, PBST was used to wash 4 times for 5 min each time. Then the membranes were incubated at 37 °C with diluted (1:10,000 (*v*/*v*)) horseradish peroxidase labeled goat anti-mouse IgG for 1 h. Finally, after washing the film 4 times, the ECL method was used to develop the image, and the gel imager was photographed.

### 2.7. Antimicrobial Activity

To detect whether rToBPI1/LBP and rToBPI2/LBP have antibacterial effects on Gram-positive bacteria (*S. agalactiae*, *S. iniae*, *S. aureus*, *C. glutamicum*) and Gram-negative bacteria (*E. coli*, *V. harveyi*, *V. parahaemolyticus* and *V. alginolyticus*), the absorbance was measured at 600 nm at different time points by using the NanoDrop 2000 [19]. The eight strains were cultured in the corresponding liquid medium overnight and then inoculated into 10 mL of the corresponding liquid medium for 10 h. After that, the bacteria were centrifugally collected and washed with 1 × PBS three times. The bacteria were precipitated and diluted to 2 × 10^8^ CFU/mL by a 10-fold dilution. rToBPI1/LBP and rToBPI2/LBP were diluted with 1 × PBS (pH = 7.4) to different concentrations (50 and 100 μg/mL); and 100 μL of the diluted rToBPI1/LBP or rToBPI2/LBP protein solution was evenly mixed with 100 μL of different bacterial suspensions. After incubation at 37 °C, the OD_600_ value was detected every 1 h. PBS mixed with the bacterial solution was used as a negative control. The experiment was repeated three times.

### 2.8. Cell Membrane Permeability Assay

Membrane permeability was measured by fluorescence staining using the Live/Dead^®^ BacLight TM Bacterial Viability Kit (Thermo) [20]. Briefly, 150 μL of rToBPI1/LBP or rToBPI2/LBP protein (100 μg/mL) was incubated for 2 h with equal volumes of *E. coli* (2.2 × 10^8^ CFU/mL), *V. alginolyticus* (2.1 × 10^8^ CFU/mL) and *S. agalactiae* (1.5 × 10^8^ CFU/mL), respectively. Then 1 μL of the fluorescent staining mixture (STYO9 0.5 μL and PI 0.5 μL) was added and incubated for 30 min away from light. The samples were observed and photographed at the excitation wavelengths of 485 nm and 535 nm and the emission wavelengths of 498 nm and 617 nm. PBS mixed with bacteria was used as a control.

### 2.9. Statistical Analyses

All real-time quantitative PCR reactions and antibacterial experiments were repeated three times and analyzed by SPSS 26.0. The statistical *p*-values were calculated by a one-way analysis of variance (One-Way ANOVA). Statistically significant differences were designated at * *p* < 0.05 and highly significant differences at ** *p* < 0.01.

## 3. Results

### 3.1. ToBPI1/LBP and ToBPI2/LBP Sequence Analysis

**Figure 1:**
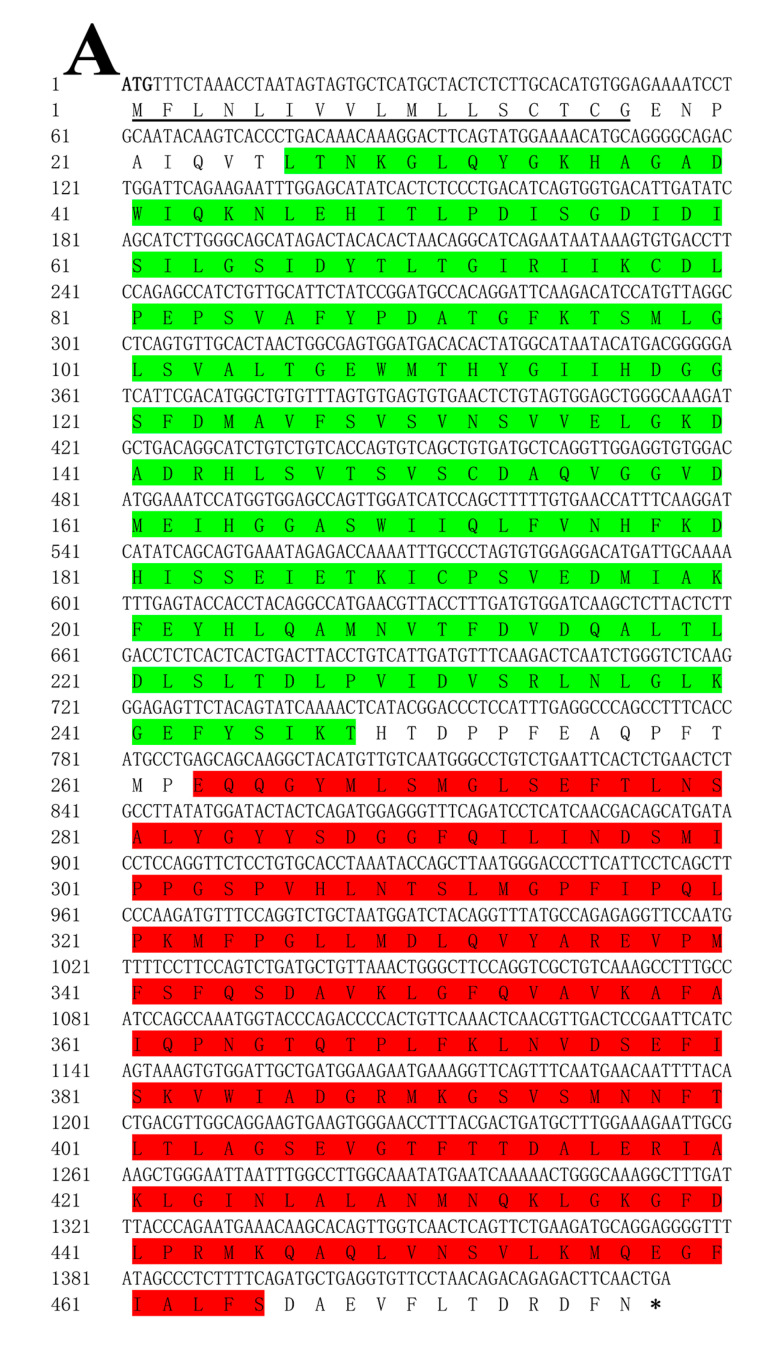

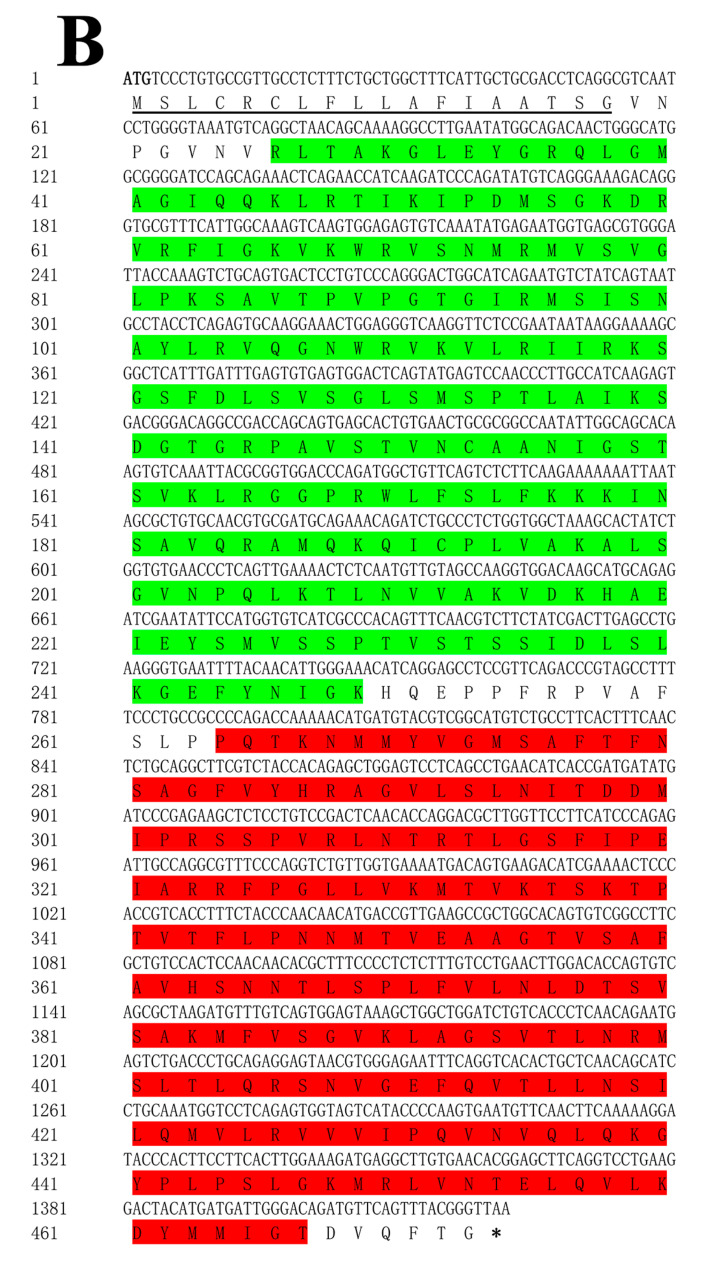
Sequence analysis of ToBPI1/LBP and ToBPI2/LBP. (**A**,**B**): nucleotide sequence and derived amino acid sequence of ToBPI1/LBP and ToBPI2/LBP. Signal peptide sequences are indicated by horizontal lines. The N-terminal domain is represented by green, and the C-terminal domain is represented by red.

**Figure 2:**
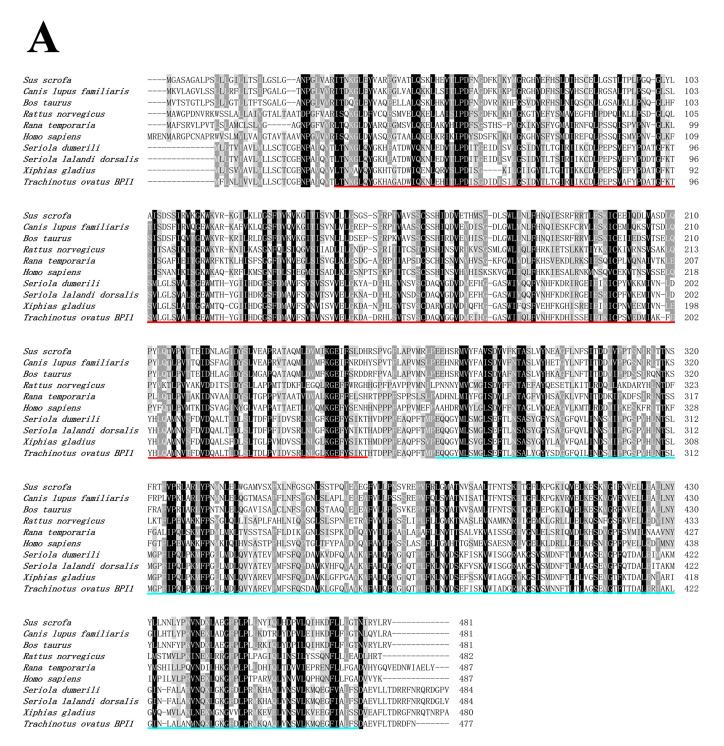

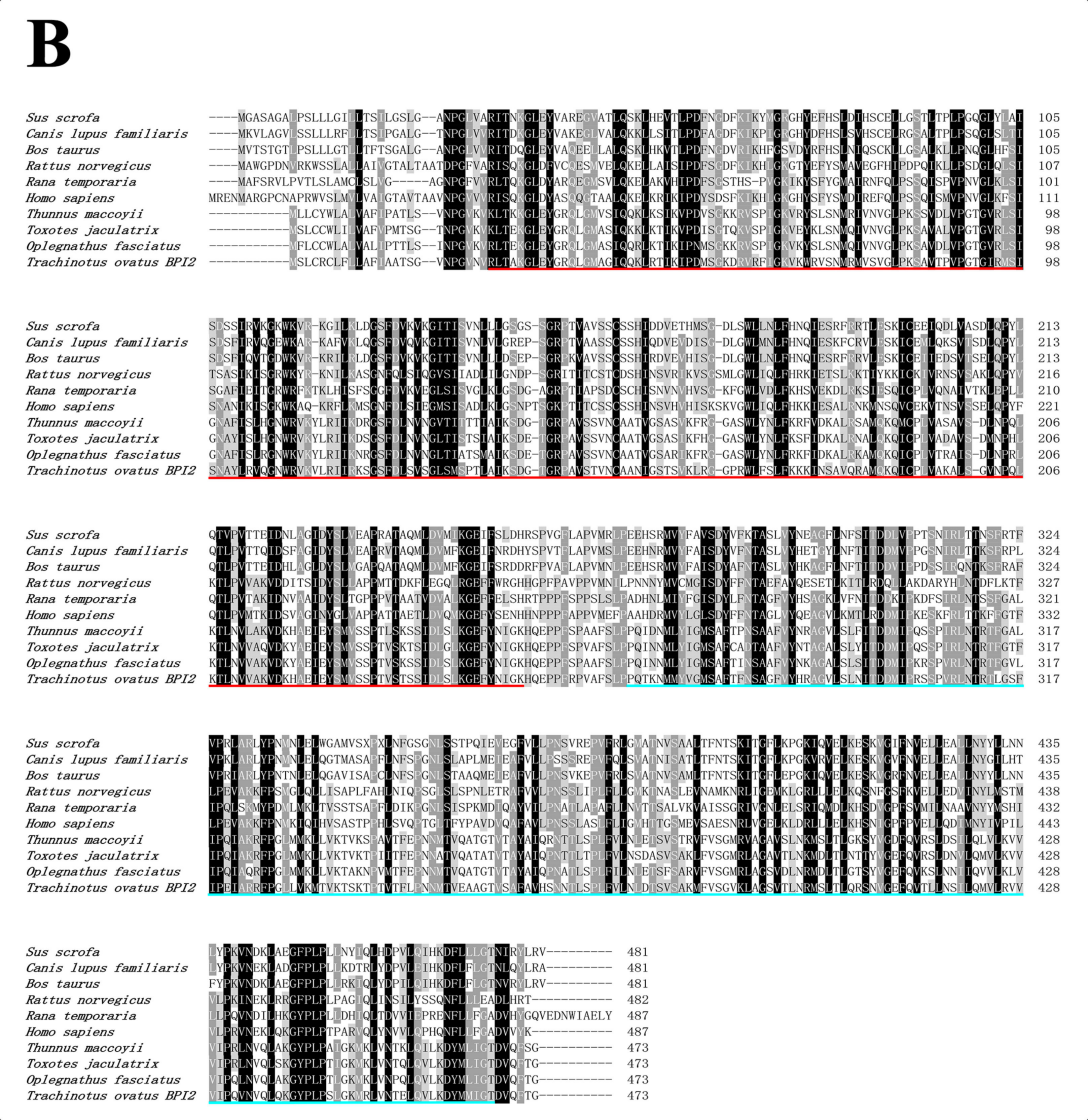
Comparison of ToBPI1/LBP (**A**) and ToBPI2/LBP (**B**) with the homologous sequences of BPI/LBP. The black part at the bottom has the same sequence, and the grey part has high repeatability. The N-terminal domains are represented by red lines and C-terminal domains by blue lines.

**Figure 3:**
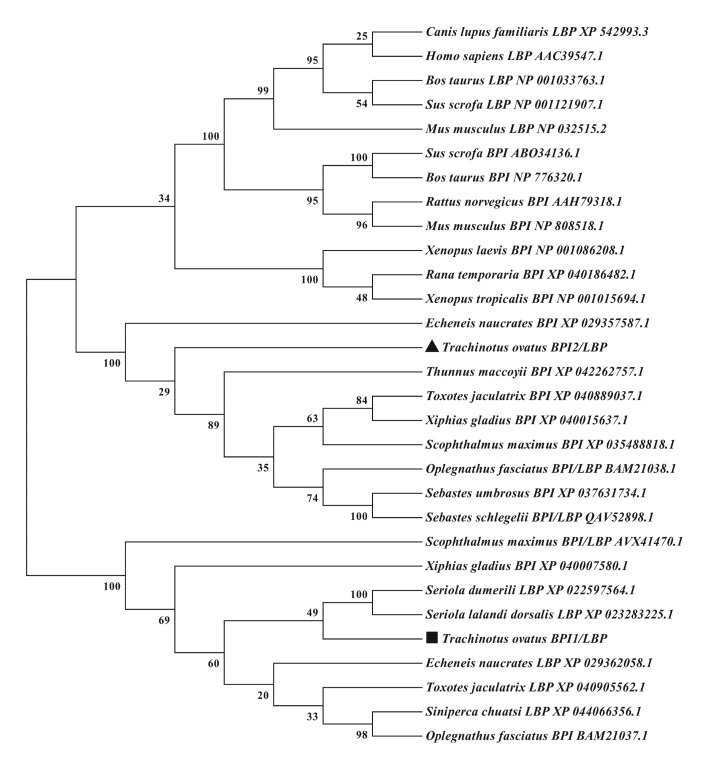
The amino acid sequences of ToBPI1/LBP and ToBPI2/LBP were compared with the known sequences of BPI, LBP and BPI/LBP from other species. The NJ method was used to construct an evolutionary tree. Boot the NJ tree 1000 times to check the repeatability of the results. ToBPI1/LBP is denoted by a black square, and ToBPI2/LBP is indicated by a black triangle.

A sequence analysis of ToBPI1/LBP and ToBPI2/LBP revealed that their ORFs were 1434 bp and 1422 bp, containing 478 and 474 amino acids with molecular weights of 52.33 kDa and 51.49 kDa, and isoelectric points of 4.77 and 10.7, respectively. The signal peptide regions of ToBPI1/LBP and ToBPI2/LBP were 1–17 and 1–18 amino acids, respectively (Figure 1A,B). Both ToBPI1/LBP and ToBPI2/LBP possessed the typical BPI/LBP protein characteristics, including an N-terminal BPI/LBP/CETP domain and a C-terminal BPI/LBP/CETP domain (Figure 1A,B). The blast analysis revealed that ToBPI1/LBP was highly consistent with the greater amberjack *Seriola dumerili* (86.16%), and ToBPI2/LBP was highly consistent with the southern bluefin tuna *Thunnus maccoyii* (71.25%). Both genes are not comparable to the mammalian sequences (Figure 2A,B). The phylogenetic tree showed that ToBPI1/LBP and ToBPI2/LBP did not converge into one branch. ToBPI1/LBP is clustered with *S. dumerili* and *S. lalandi dorsalis*, while ToBPI2/LBP is distant from other fish, and both genes were far away from other species (Figure 3).

### 3.2. Tissue Distribution of ToBPI1/LBP and ToBPI2/LBP in Healthy T. ovatus

**Figure 4:**
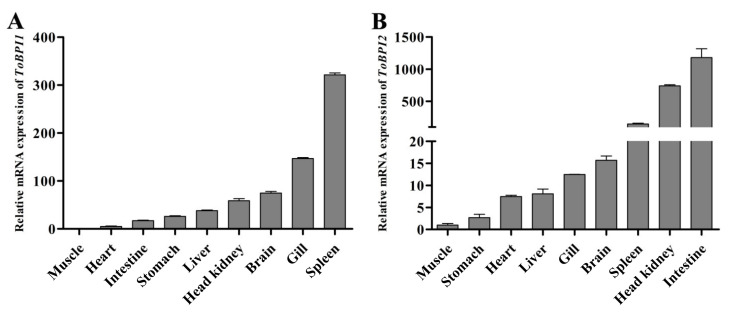
The expression of *ToBPI1/LBP* (**A**) and *ToBPI2/LBP* (**B**) in healthy tissues of *T. ovatus* was detected by real-time quantitative PCR. For comparison, the expression level in muscles was set at 1.0. Each T-line represents the mean standard deviation (*n* = 3).

Both genes were found to be expressed in all tissues. The spleen had the highest levels of *ToBPI1/LBP* expression, while the gill, brain, cephalic kidney, liver, stomach, intestine, and heart had lower levels (Figure 4A). *ToBPI2/LBP* expression was highest in the intestine, followed by the head, kidney and spleen, and lowest in the brain, gill, liver, heart and stomach (Figure 4B).

### 3.3. Expression of ToBPI1/LBP and ToBPI2/LBP in Different Tissues after Bacterial Infection

**Figure 5:**
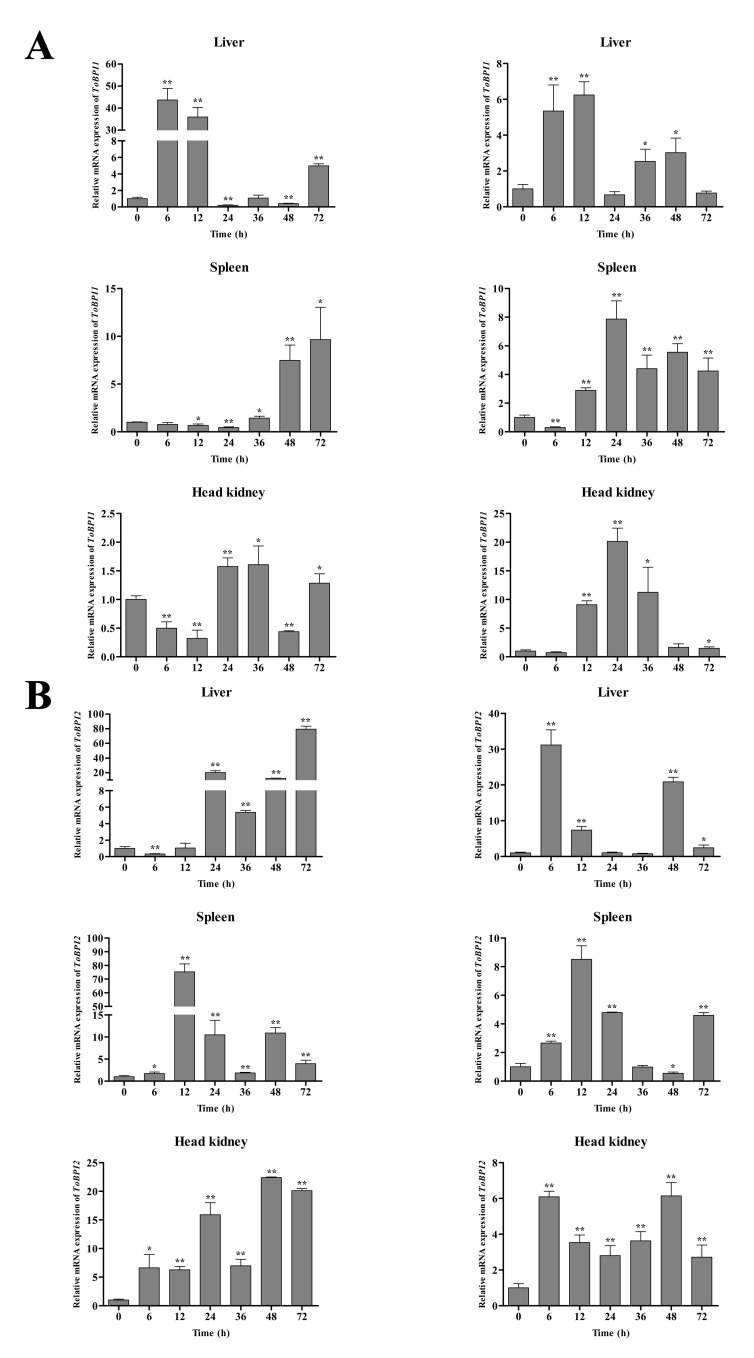
The expressions of ToBPI1/LBP (**A**) and ToBPI2/LBP (**B**) in tissues of *T. ovatus* were detected by quantitative real-time PCR at different time points after infection with *V. alginolyticus* (left) and *S. agalactiae* (right). For comparison, the expression level at 0 h for each group was set as 1. Each T-line represents the mean standard deviation (*n* = 3). There was no significant difference *p* > 0.05. The difference was considered significant when * *p* < 0.05, highly significant when ** *p* < 0.01.

In the liver, the expression of ToBPI1/LBP and ToBPI2/LBP peaked at 6 h and 72 h, respectively, following the infection with *V. alginolyticus* (Figure 5). In the spleen, ToBPI1/LBP showed a gradual up-regulation trend and peaked at 72 h. At 12 h, ToBPI2/LBP reached a peak and began to decline. ToBPI1/LBP and ToBPI2/LBP peaked in the head and kidney at 36 h and 48 h, respectively. In the *S. agalactiae* group, ToBPI1/LBP and ToBPI2/LBP peaked in the liver after infection at 12 h and 6 h, respectively. ToBPI1/LBP and ToBPI2/LBP both tended to decline in the spleen after reaching peaks at 24 h and 12 h, respectively. ToBPI1/LBP and ToBPI2/LBP peaked in the head and kidney at 24 h and 48 h, respectively. These findings implied that ToBPI1/LBP and ToBPI2/LBP played a role in *T. ovatus’s* immunological protection against bacterial invasion.

### 3.4. Recombinant Expression and Purification of rToBPI1/LBP and rToBPI2/LBP

**Figure 6:**
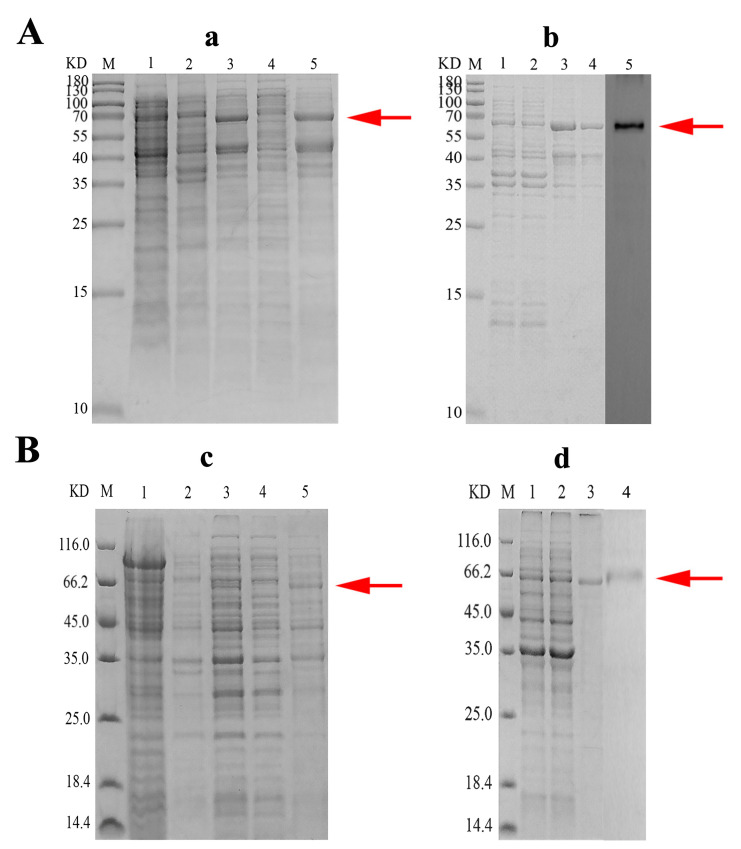
Expression and purification of rToBPI1/LBP (**A**) and rToBPI2/LBP (**B**); (**a**,**c**): Lane M: Protein molecular standard; Lane 1: pSumo-mut and pET-32a no-load plasmid; Lane 2: rToBPI1/LBP and rToBPI2/LBP without IPTG induction; Lane 3: rToBPI1/LBP and rToBPI2/LBP after 4 h induction by IPTG; Lane 4: Supernatant in rToBPI1/LBP and rToBPI2/LBP lysate; Lane 5: Precipitation in rToBPI1/LBP and rToBPI2/LBP lysate. (**a**,**c**): Lane M: Protein molecular standard; Lane 1: pSumo-mut and pET-32a no-load plasmid; Lane 2: rToBPI1/LBP and rToBPI2/LBP without IPTG induction; Lane 3: rToBPI1/LBP and rToBPI2/LBP after 4 h induction by IPTG; Lane 4: Supernatant in rToBPI1/LBP and rToBPI2/LBP lysate; Lane 5: Precipitation in rToBPI1/LBP and rToBPI2/LBP lysate. (**b**): Lane M: Protein molecular standard; Lane 1: Precipitation in rToBPI1/LBP lysate; Lane 2: Pre-flow purified solution; Lane 3–4: Recombinant rToBPI1/LBP purified using purified column; Lane 5: Western blot of rToBPI1/LBP; (**d**): Lane M: Protein molecular standard; Lane 1: Precipitation in rToBPI2/LBP lysate; Lane 2: Pre-flow purified solution; Lane 3: Recombinant rToBPI2/LBP purified using purified column; Lane 4: Western blot of rToBPI2/LBP;.

Results bands of about 64.6 kDa and 66.6 kDa were seen (Figure 6a,c), which is consistent with the expected molecular weight results, while no band was found in the non-IPTG induced bacterial solution. The findings in Western blotting revealed a single strip (Figure 6b,d), indicating that it could react specifically with rToBPI1/LBP and rToBPI2/LBP.

### 3.5. Antimicrobial Activity

**Figure 7:**
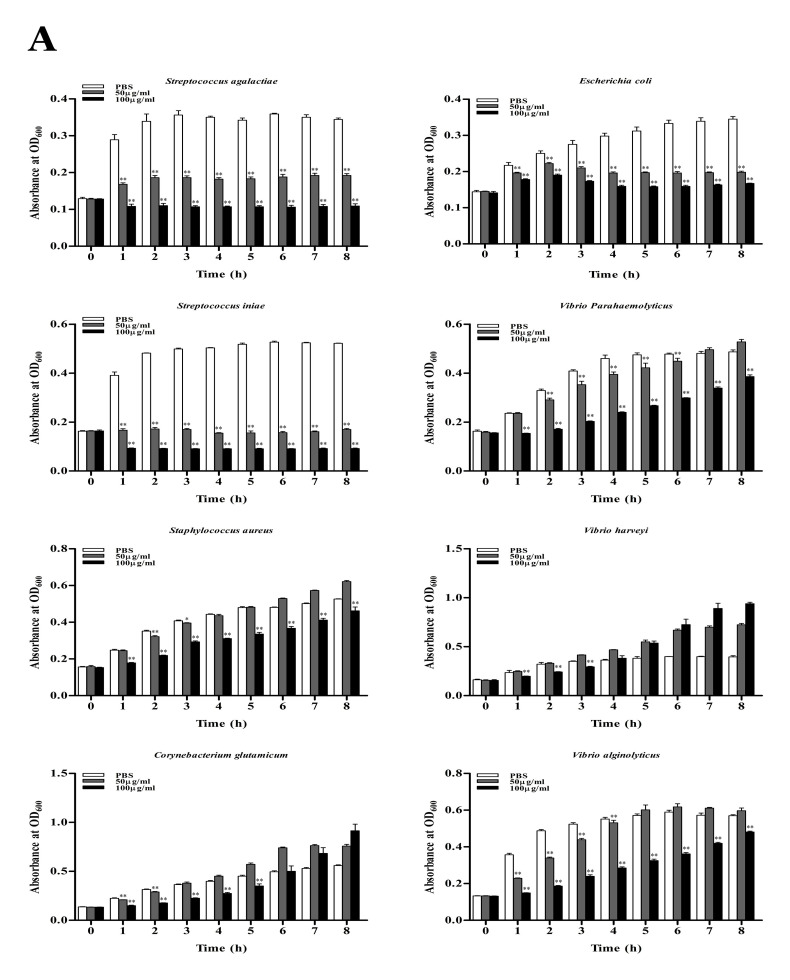

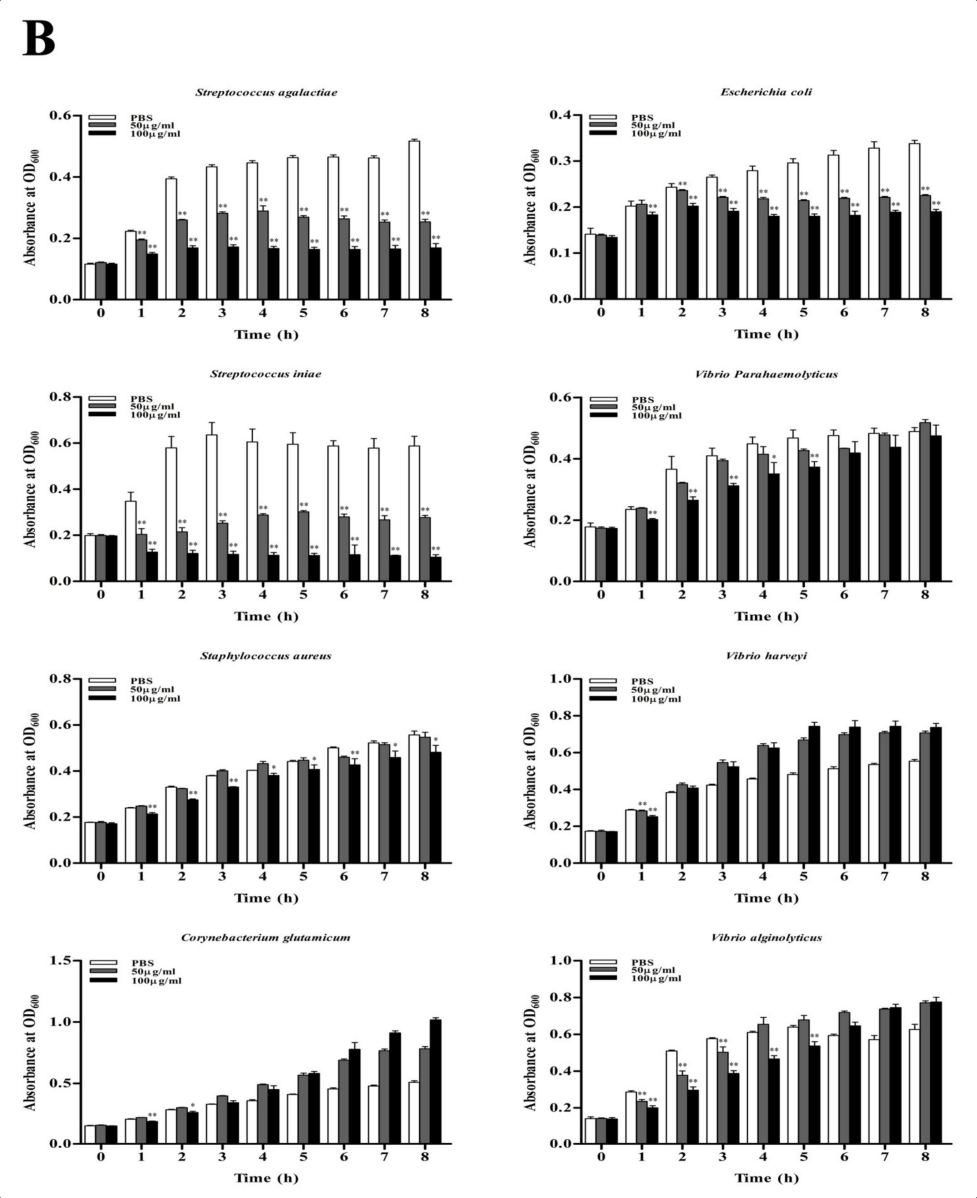
Bacteriostasis of rToBPI1/LBP (**A**) and rToBPI1/LBP (**B**) against Gram-positive bacteria *S. agalactiae*, *S. iniae*, *S. aureus* and *C. glutamicum* as well as Gram-negative bacteria *E. coli*, *V. parahaemolyticus*, *V. harveyi* and *V. alginolyticus* at 50 μg/mL and 100 μg/mL. Gram-positive bacteria are on the left panel, whereas Gram-negative bacteria are on the right. The data shown are the average of three independent experiments. The difference was significant when * *p* < 0.05, highly significant when ** *p* < 0.01.

To test the bacteriostatic activity of rToBPI1/LBP and rToBPI2/LBP, the OD_600_ of Gram-negative bacteria (*E. coli*, *V. alginolyticus*, *V. parahaemolyticus* and *V. harveyi*) and Gram-positive bacteria (*S. aureus*, *S. agalactiae*, *S. iniae* and *C. glutamicum*) were measured at various time points (Figure 7A,B). The results demonstrated that, at two doses, rToBPI1/LBP and rToBPI2/LBP significantly inhibited the growth of *E. coli*, *S. agalactiae* and *S. iniae*. The bacteriostatic impact on *S. aureus*, *C. glutamicum*, *V. parahaemolyticus*, *V. harveyi* and *V. alginolyticus* all reduced over time when rToBPI1/LBP concentration was 100 μg/mL. The RToBPI1/LBP protein had no antibacterial effects on *C. glutamate* and *V. harveyi* after 6 h and 4 h, respectively. When the concentration of rToBPI1 was 50 μg/mL, there was almost no bacteriostatic effect on *S. aureus*, *C. glutamicum* and *V. harveyi*, and the effect on *V. parahaemolyticus* and *V. alginolyticus* was subpar (Figure 7A). The bacteriostatic effect on *S. aureus*, *V. parahaemolyticus* and *V. alginolyticus* was obtained at a concentration of 100 μg/mL rToBPI2/LBP, and the bacteriostatic impact waned over time. The RToBPI2/LBP protein had an antibacterial effect on *C. glutamicum* and *V. harveyi* only at 2 h and 1 h. When the concentration of rToBPI2/LBP was 50 μg/mL, there was no bacteriostatic effect on *S. aureus*, *C. glutamicum*, *V. parahaemolyticus* and *V. Harveyi*; there was a bacteriostatic effect on *V. alginolyticus* only in the first 3 h (Figure 7B).

### 3.6. Permeability of Cell Membrane

**Figure 8:**
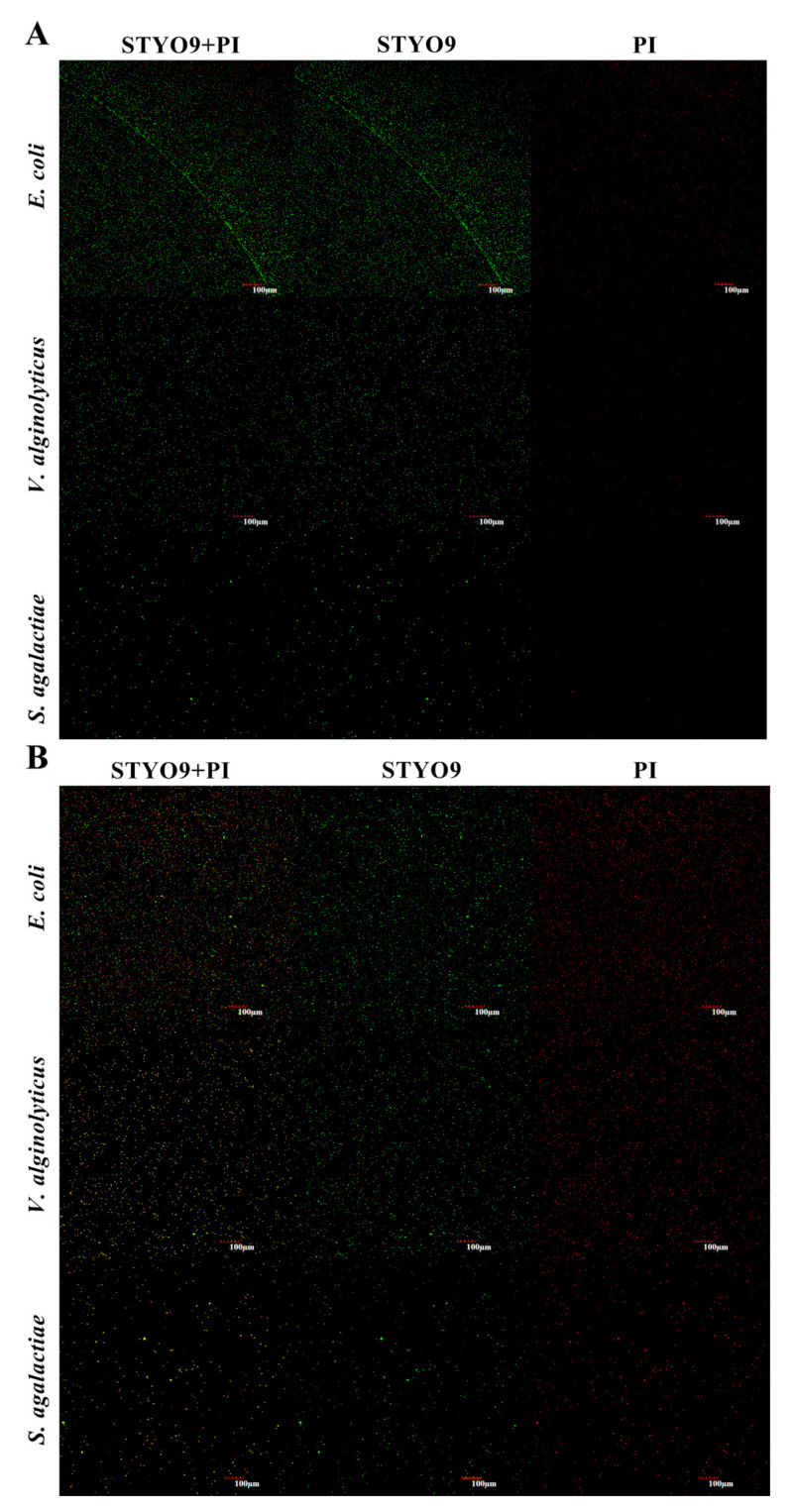

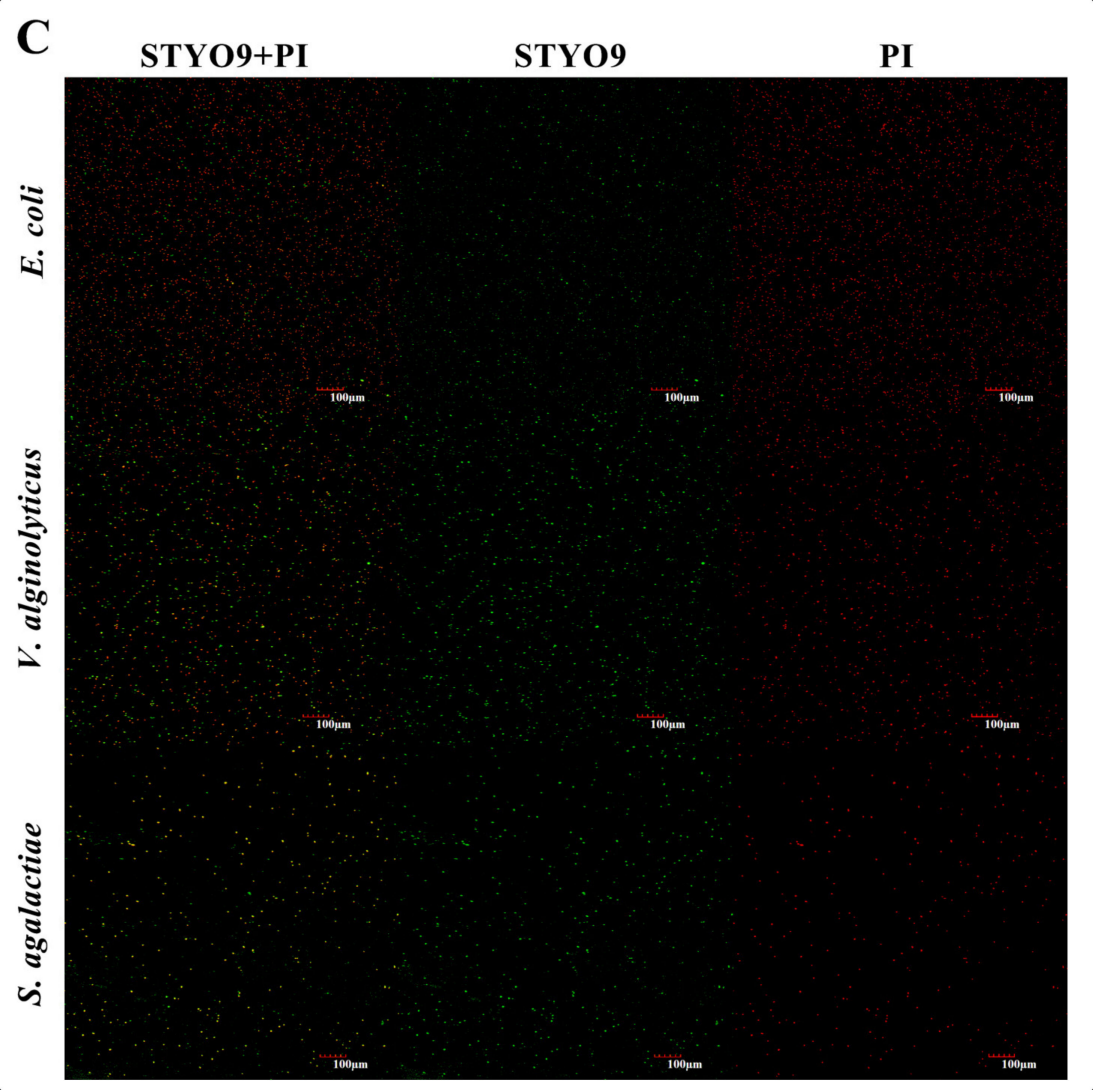
*E. coli*, *V. alginolyticus* and *S. agalactiae* treated with rToBPI1/LBP and rToBPI2/LBP proteins were used to observe the permeability of bacterial cell membrane. PBS was used as a control. (**A**) the bacteria treated with PBS; (**B**) rToBPI1/LBP treated bacteria; (**C**) rToBPI2/LBP treated bacteria; STYO9 is shown in green, and PI is shown in red.

The results of fluorescence staining showed that after rToBPI1/LBP and rToBPI2/LBP protein treatment, the red fluorescence (PI) significantly increased (Figure 8B,C), while the PI in the PBS control group did not change (Figure 8A).

## 4. Discussion

The BPI/LBP antimicrobial peptide is crucial for the host’s resistance to bacterial infection, which was also found in many teleost fish species, and the amount is species-specific [21,22]. In this study, two new BPI/LBP genes (*ToBPI1/LBP* and *ToBPI2/LBP)* were cloned from *T. ovatus*. The amino acid sequence analysis showed they had an N-terminal BPI/LBP/CETP domain and a C-terminal BPI/LBP/CETP domain. According to previous studies, the C-terminal domain may be involved in facilitating LPS binding, while the N-terminal domain is thought to be involved in bactericidal activity and neutralizing LPS [23]. This may be due to the N-terminal domain of BPI/LBP having abundant positively-charged amino acid residues that interact electrostatically with the negatively-charged group of LPS to facilitate the binding of BPI/LBP and LPS [24]. Other bony fish, including blunt snout bream *M. amblycephala* [3], black rockfish *S. schlegelii* [12] and Japanese flounder *Paralichthys olivaceus* [25], have been shown to have identical N-terminal domains and C-terminal domains which perform similar functions.

The present study evaluated the spatial expression of *ToBPI1/LBP* and *ToBPI2/LBP* in healthy tissues of golden pompano. The results showed that *ToBPI1/LBP* was highly expressed in the brain, gill, and spleen, while *ToBPI2/LBP* was highly expressed in the spleen, cephalic kidney, and intestine (Figure 4). In other fish, BPI/LBP also showed high expression in these immune-related organizations. In *M. amblycephala*, BPI/LBP is highly expressed in the kidney [3]. BPI/LBP is highly expressed in the spleen and kidney in *Oplegnathus fasciatus* [6]. In *S. maximus*, BPI/LBP is highly expressed in the spleen [14]. ToBPI1/LBP expression was the highest in the spleen, which resembled the results of *S. schlegelii* [12]. The spleen is considered the major immune organ for the generation of adaptive immune responses [26,27], which is regarded as a crucial immunological organ in bony fish because it has lymphocytes and macrophages that can initiate immune responses [28]. The intestine, followed by the head, kidney and spleen, all had the highest levels of *ToBPI2/LBP* expression. As a crucial mucosal immunological organ of fish, the intestinal tract serves as a fish’s defense against the external environment. It is essential for fending against pathogen invasion and preventing the entry of microbial and bacterial toxins into the systemic circulation. We speculate that the differential expression of *ToBPI1/LBP* and *ToBPI2/LBP* in healthy tissues may be related to their interaction with *T. ovatus* defense against pathogen invasion in a complex aquatic environment, but further research is required to confirm this hypothesis. 

After infection with *S. agalactiae* and *V. alginolyticus*, the expressions of *ToBPI1/LBP* and *ToBPI2/LBP* in the liver, head, kidney and spleen increased considerably. Similarly, the head, kidney and spleen of *S. schlegelii* had much higher levels of the BPI/LBP homolog after being exposed to the Gram-positive bacterium *S. iniae* [12]. *P. olivaceus* also demonstrated a similar pattern in the spleen, head, kidney, intestine, gill, and liver after exposure to *S. iniae* [25]. These findings suggest that *ToBPI1/LBP* and *ToBPI2/LBP* are involved in the immune defense against pathogen invasion in *T. ovatus*.

Numerous investigations have demonstrated that BPI/LBP has strong antibacterial activity against Gram-negative bacteria but none against Gram-positive bacteria at this time [12,17]. In this study, rToBPI1/LBP and rToBPI2/LBP proteins were also confirmed to have significant inhibitory effects on the growth and proliferation of Gram-negative bacteria, which is consistent with the results of *S. schlegelii*, *C. semilaevis* and *Paa yunnanensis* [12,13,29]. The difference is that the rToBPI1/LBP and rToBPI2/LBP proteins also showed a significant antibacterial effect against the Gram-positive bacteria *S. agalactiae* and *S. iniae*. It has been discovered in earlier studies that the recombinant protein made from the human BPI N-terminal analog can inhibit the growth of the L-form *S. aureus* [30], and our study findings are compatible with these results. 

According to various studies, LPS is one of the primary components of Gram-negative bacteria’s cell walls, and BPI can damage cell membranes by binding to LPS in Gram-negative bacteria [31], eventually killing the bacteria. Recombinant ToBPI1/LBP and ToBPI2/LBP proteins were found to increase the permeability of Gram-negative bacterial and Gram-positive bacterial cell membranes in a laboratory experiment. Additionally, it was discovered while researching *C. semilaevis,* that *Pseudomonas fluorescens* treated with recombinant CsBPI caused a considerable increase in membrane permeability [13]. However, no fish studies have demonstrated that BPI/LBP directly eliminates Gram-positive bacteria. Similarly, no research has shown that BPI/LBP can harm cell membranes and increase membrane permeability in Gram-positive bacteria. In some investigations, Lipoteichoic acid (LTA), a product of Gram-positive bacteria’s cell walls, has also been discovered to be a new BPI ligand [32]. Studies have shown that both LTA and LPS use N-terminal lipid binding pockets when competing for binding to BPI. It can bind hydrophobic acyl chains, and acyl chains are dependent on BPI and LTA binding, just as they are crucial to the binding of BPI and LPS [32]. Therefore, we speculate that BPI/LBP may interact with LTA to affect the cell membrane of Gram-positive bacteria. However, more research is required to determine the underlying mechanism by which the recombinant ToBPI1/LBP and ToBPI2/LBP proteins affect Gram-positive bacteria. 

## 5. Conclusions

In conclusion, the ORF sequences of *ToBPI1* and *ToBPI2* were successfully cloned at 1434 bp (478 aa) and 1422 bp (474 aa) in length, respectively. A protein domain prediction analysis showed that ToBPI1 and ToBPI2 both contained an N-terminal signal peptide, an N-terminal domain and a C-terminal domain. The homology comparison showed that ToBPI1/LBP was highly consistent with *Seriola dumerili* (86.16%), and ToBPI2/LBP was highly consistent with *Thunnus maccoyii* (71.25%). A phylogenetic analysis showed that ToBPI1 and ToBPI2 were not clustered together. Real-time PCR analysis showed that *ToBPI1* and *ToBPI2* were expressed at the highest levels in the spleen and intestine of healthy golden pompano, respectively. The expression levels of ToBPI1 and ToBPI2 in immune-related tissues were significantly upregulated after stimulation with *S. agalactiae* and *V. alginolyticus*. In vitro bacteriostasis tests showed that rToBPI1 and rToBPI2 could effectively inhibit the growth of *E. coli*, *S. agalactiae* and *V. alginolyticus*. Cell membrane permeability experiments showed that rToBPI1 and rToBPI2 could increase bacterial cell membrane permeability. The present findings offer new perspectives and may serve as important references for the immune defense effect of *ToBPI1/LBP* and *ToBPI2/LBP* in *T. ovatus*.

## Figures and Tables

**Table 1:** Primers used in this study.

Primers Name	Sequence 5′–3′	Purpose
ToBPI1/LBP -F	ATGTTTCTAAACCTAATAGTAGTGCTCATGC	Cloning
ToBPI1/LBP -R	TCAGTTGAAGTCTCTGTCTGTTAGG	Cloning
ToBPI2/LBP -F	ATGTCCCTGTGCCGTTG	Cloning
ToBPI2/LBP -R	TTAACCCGTAAACTGAACATCTGTC	Cloning
ToBPI1/LBP -F	CCTTTGCCATCCAGCCAAAT	Real-time PCR
ToBPI1/LBP -R	CCACTTCACTTCCTGCCAAC	Real-time PCR
ToBPI2/LBP -F	GCCCACAGTTTCAACGTCTT	Real-time PCR
ToBPI2/LBP -R	GTGTTGAGTCGGACAGGAGA	Real-time PCR
β-action-F	TACGAGCTGCCTGACGGACA	Real-time PCR
β-action-R	GGCTGTGATCTCCTTCTGC	Real-time PCR
mToBPI1 LBP-F	CGCGGATCCGAAAATCCTGCAATACAAGTC	Prokaryotic expression
mToBPI1/LBP-R	CCGCTCGAGGTTGAAGTCTCTGTCTGTTAGG	Prokaryotic expression
mToBPI2/LBP-F	CGGAATTCGTCAATCCTGGGGTAAATGT	Prokaryotic expression
mToBPI2/LBP-R	CCCTCGAGACCCGTAAACTGAACATCTGT	Prokaryotic expression

## Data Availability

The data presented in this study are available in the article.
